# Involvement of Allelopathy in the Invasive Potential of *Tithonia diversifolia*

**DOI:** 10.3390/plants9060766

**Published:** 2020-06-19

**Authors:** Hisashi Kato-Noguchi

**Affiliations:** Department of Applied Biological Science, Faculty of Agriculture, Kagawa University, Miki, Kagawa 761-0795, Japan; kato.hisashi@kagawa-u.ac.jp; Tel.: +81-87-891-3086

**Keywords:** allelopathy, invasive plant, phytotoxicity, sesquiterpene lactone, *Tithonia diversifolia*

## Abstract

*Tithonia diversifolia* (Hemsl.) A. Gray (Asteraceae) is native to Mexico and Central America. The species is spreading quickly and has naturalized in more than 70 countries. It has often been recorded as a harmful invasive plant that disturbs native plant communities. Phytotoxic chemical interactions such as allelopathy between invasive plants and native plants have been reported to play an important role in the invasion. Evidence for allelopathy of *T. diversifolia* has accumulated in the literature over 30 years. Thus, the objective of this review was to discuss the possible involvement of allelopathy in the invasive potential of *T. diversifolia.* The extracts, root exudates, and plant residues of *T. diversifolia* inhibited the germination and growth of other plant species. The soil water and soil collected from *T. diversifolia* fields also showed inhibitory growth effects. The decomposition rate of *T. diversifolia* residues in soil was reported to be high. Phytotoxic substances such as sesquiterpene lactones were isolated and identified in the extracts of *T. diversifolia*. Some phytotoxic substances in *T. diversifolia* may be released into the soil through the decomposition of the plant residues and the exudation from living tissues of *T. diversifolia*, including its root exudates, which act as allelopathic substances. Those allelopathic substances can inhibit the germination and growth of neighboring plants and may enhance the competitive ability of the plants, make them invasive.

## 1. Introduction

*Tithonia diversifolia* (Hemsl.) A. Gray (phylum: Spermatophyta, class: Dicotyledonae, order: Asterales, family: Asteraceae, genus: Tithonia) is known as Mexican sunflower, tree marigold, or Nitobe chrysanthemum. It grows rapidly, reaching 2–3 m in height with large alternate lobe leaves (up to 45 cm long). The monocarpic capitulums are 10–30 cm long and bear bright yellow flowers (5–15 cm in diameter). The plant often forms pure stands with high density (8–20 plants/m^2^) [1,2,3].

*T. diversifolia* can be harvested year-round and all parts of the plants have been used by indigenous people as folk medicine for a wide range of diseases and aliments, through topical administration to treat abdominal pain, wounding, dermatosis, and muscular disorder; and through oral administration to treat infection, malaria, fever, hepatitis, and diabetes [3,4]. Thus, the plants have a broad spectrum of medicinal values.

More than a hundred secondary metabolites in many classes have been isolated from various parts of *T. diversifolia* extracts, including sesquiterpenoids, diterpenoids, and flavonoids. Tagitinins A, C, and F were first isolated from *T. diversifolia* [5,6]. The effects of the extracts of *T. diversifolia* and those compounds isolated from *T. diversifolia* have been widely studied in human cell lines, microorganisms, and some animal models. These studies showed an extended spectrum of biological activities for the extracts and compounds, such as anti-inflammatory and analgesic activities; antiprotozoal activity, including antimalarial effects; and antiviral and anticancer activities. The compounds of *T. diversifolia* and their pharmacological activities have been discussed in the review articles [3,4,7]. Therefore, *T. diversifolia* is one of the important sources of pharmacologically active substances, and the study of these compounds may contribute to developing potential medicines for various treatments.

*T. diversifolia* also works as green manure, increasing crop productivity, and acts as fodder for domestic animals because of its high mineral and nutrient values [8,9,10,11]. On the other hand, *T. diversifolia* aggressively expands its habitat into agricultural and non-agricultural areas, becoming a serious farmland weed and disturbing native plant communities as an invasive plant species [2,7,12]. The species has shown allelopathic potency on the germination and growth of several other plant species [13,14,15]. Allelopathy may play an important role in the invasion of *T. diversifolia*. The objective of this review was to discuss the possible involvement of allelopathy in the invasive potential of *T. diversifolia.* Thus, this review summarized the allolopathic properties and invasive traits of *T. diversifolia* and discussed the importance of allelopathy for its invasive characteristics.

## 2. Allelopathic Property of *T. diversifolia*

### 2.1. Extract of T. diversifolia

The aqueous shoot extracts of *T. diversifolia* were applied onto the soil after three woody plant species, *Monodora tenuifolia* Benth., *Dialium guineense* Willd. and *Hildegardia barteri* Mast. had been planted, and the effects of the treatments were evaluated after 10 weeks. The extract treatments resulted in reductions in the shoot length, leaf area and number, and chlorophyll content of all of the woody plants [16]. The aqueous leaf extracts (10%, *w/v*) were applied onto the field soil (10 L/ha) of the cowpea (*Vigna unguiculata* (L.) Walp.) cropping system. The treatment showed a 63.7% reduction in the total weed density in the field at 65 days after the treatment. However, the treatment increased the cowpea grain yield by 71.2% because of the suppression of the weeds [17]. In addition, the aqueous shoot extracts of *T. diversifolia* extract-dependently suppressed the growth of radicles and plumules of maize (*Zea mays* L.) seedlings [18], the germination of lettuce (*Lactuca sativa* L.) and *Bidens pilosa* L. [19], and the germination and growth of *Tridax procumbens* L. [20].

Four aqueous extracts of *T. diversifolia*, taken from both fresh and dry shoots and roots, inhibited the germination and growth parameters, such as the plant height, root length, leaf area, and plant weight of rice (*Oryza sativa* L.) and *Amaranthus cruentus* L. The effectiveness of the fresh shoot extract was the greatest, followed by the dry shoot, fresh root, and dry root extracts [21,22]. Aqueous extracts of both green leaves and senescent leaves of *T. diversifolia* suppressed the germination and seedling growth of 12 crop plants and 5 weed species (Table 1). However, there was no difference in the inhibitory activity between the extracts of the green leaves and senescent leaves [23]. It was also found that water stress treatments for *T. diversifolia* enhanced the inhibitory effects of its aqueous root extracts [13].

Ethyl acetate extracts of whole plants of *T. diversifolia* inhibited the germination and seedling growth of tomato (*Lycopersicon esculentum* L.), onion (*Allium cepa* L.), lettuce, and cress (*Lepidium sativum* L.) [14]. Aqueous methanol extracts of *T. diversifolia* leaves also suppressed the seedling growth of *Lolium multiflorum* L., *Phleum pratense* L., *Echinochloa crus-galli* (L.) P.Beauv. and cress [15]. *T. diversifolia* leaves were extracted with water only, a mixture of water and methanol (1:1, *v/v*), and methanol only, and the inhibitory activities of these extracts were compared against the growth of *Sorghum bicolor* (L.) Moench. The methanol extract showed the greatest inhibitory effect, followed by the solvent mixture extract and the water extract [24]. The inhibitory effects of methanol extracts of *T. diversifolia* shoots on the growth of cowpea were also reported, which were greater than those of their aqueous extracts [25]. The findings described in this section indicate that the extracts of *T. diversifolia* have inhibitory effects on the germination and growth of several other plant species, and probably contain some phytotoxic substances, which may act as allelopathic substances. The compounds in *T. diversifolia* can be extracted with water and organic solvents, and are more extractable with methanol than water.

In some cases, the extracts of *T. diversifolia* increased the plant growth and productivity of other plants. Aqueous extracts of *T. diversifolia* shoots enhanced the germination and plant growth of cowpea [26], and increased the leaf area and weight of maize plants that were older than two weeks of age from germination [17]. Aqueous extracts (10%, *w/v*) of *T. diversifolia* leaves were applied either directly onto cultivated soil containing beans (*Phaseolus vulgaris* L.) or as foliar spray. Both treatments enhanced the growth and yield of the beans. The enhancements were considered to be consequences of the increasing the concentrations of chlorophyll and metabolites in the bean plants, such as phenylalanine and tryptophan [27]. The extracts and residues of *T. diversifolia* also worked as fertilizers because of their high mineral values [8,10,11].

### 2.2. Root Exudate of T. diversifolia

*T. diversifolia* was grown in plastic pots and its root exudates with the capillary water were drained from holes in the bottoms of the pots and accumulated. The accumulated root exudates of *T. diversifolia* suppressed the germination, shoot length, and leaf area of *Amaranthus dubius* Mart. ex Thell. [28]. The inhibitory effects indicate that some allelopathic substances, which have not yet been identified, were probably released into the soil, as the root exudates from *T. diversifolia*, and suppressed the germination and growth of *A. dubius*.

### 2.3. Residue of T. diversifolia

The leaves of *T. diversifolia* were mixed with soil and okra (*Abelmuscus esculentus* (L.) Moench) seeds were sown into the soil. The treatments resulted in the suppression of the germination and growth of okra. However, the treatments with high concentrations of the leaf mixtures (100 g /pot) resulted in the enhancement of the vegetative and reproductive growth of okra because of high mineral contents in the leaves [29]. The leaf residues of *T. diversifolia* also inhibited the growth of rice seedlings [30]. Those results indicate that unidentified allelopathic substances may be released into the soil by the decomposition of the leaf residues of *T. diversifolia*.

### 2.4. T. diversifolia Field Soil

Soil collected from *T. diversifolia* fields suppressed the emergence of 5 weed species, namely *Ancanthospermum hispidium* D.C., *Bidens pilosa* L., *Euphorbia heterophylla* L., *Panicum masimum* Jacq., and *Pennisetum polysachion* (L.) Schult. [31]. Soil collected from *T. diversifolia* fields also inhibited the seedling growth of rice, radish (*Raphanus sativa* L.), *Sorghum bicolor* (L.) Moench, *Digitaria cliaris* (Retz.) Koel, *Cyperus iria* L., and *Amarantthus viridis* L. [13,30]. In addition, the soil water separated from the soil collected from *T. diversifolia* fields inhibited the seedling growth of rice, radish, *S. bicolor*, *D. cliaris*, *C. iria*, and *A. viridis* [30]. Those findings suggest that some allelopathic substances had accumulated in the field soils. The accumulation may occur through the exudation of those substances from living plant tissues of *T. diversifolia* and/or through the decomposition of the plant residues in the soils. Allelopathic activities of the extracts, root exudate, residues, field soils, and soil water of *T. diversifolia* are summarized in Table 1.

**Table 1:** Allelopathic activities of the extracts, root exudate, residues, field soils, and soil water of *T. diversifolia* on target plant species.

Source	Inhibitory Activity	Target Plant Species and Reference
Aqueous extract of shoot	Germination	Lettuce, *Bidens pilosa* [19]
	Growth	Maize [18]
	Germination, growth	*Tridax procumbens* [20]
	Growth, chlorophyll	*Monodora tenuifolia*, *Dialium guineense*, *Hildegardia barteri* [16]
Aqueous extract of shoot and root	Germination, growth	Rice, *Amaranthus cruentus* [21,22]
Aqueous extract of leaf	Germination, growth	Barley, cabbage, cucumber, lettuce, mung bean oat, onion, radish, rice, *Sorghum bicolor*, tomato, wheat, *Digitaria adscendens*, *Rottboellia exaltata*, *Aeschynomene americana*, *Cyperus iria*, *Amaruntus viridis* [23]
	Weed density	Field weeds [17]
Aqueous methanol extract of leaf	Growth	Cress, *Lolium multiflorum*, *Phleum pratense, Echinochloa crus-galli* [15]
Aqueous and methanol extract of leaf	Growth	*Sorghum bicolor* [24]
Aqueous and methanol extract of shoot	Growth	Cowpea [25]
Ethyl acetate extract of whole plant	Germination, growth	Cress, lettuce, tomato, onion [14]
Root exudate	Germination, growth	*Amaranthus dubius* [28]
Leafe residue	Growth	Rice [30]
	Germination, growth	Okra [29]
Field soil of *T. diversifolia*	Germination	*Ancanthospermun hispidium, Bidens pilosa, Euphorbia heterophylla, Panicum masimum, Pennisetum polysachion* [31]
	Germination, growth	Rice, radish, *Sorghum bicolor*, *Digitaria cliaris*, *Cyperus iria, Amaruntus viridis* [13,30]
Soil water from field soil	Germination, growth	Rice, radish, *Sorghum bicolor*, *Digitaria cliaris*, *Cyperus iria, Amaruntus viridis* [30]

## 3. Allelopathic Substances of *T. diversifolia*

Two sesquiterpene lactones (tagitinin A and tagitinin C) and a flavonoid (hispidulin) were isolated from aerial parts of *T. diversifolia.* These compounds showed inhibitory effects on the germination of radish, cucumber (*Cucumis sativus* L.), and onion [32] (Table 2). Fourteen compounds, including 12 sesquterpene lactones, were isolated from ethyl acetate extracts of whole plants of *T. diversifolia,* 11 compounds from which suppressed the coleoptile growth of wheat (*Triticum aestivum* L.). Of these, 1β-methoxydiversifolin, tagitinin A, and tagitinin C were major compounds in the extracts, which inhibited the seedling growth of tomato, onion, lettuce, and cress in a concentration-dependent manner [14].

The aqueous methanol extract of *T. diversifolia* leaves was separated by a bioassay-guided purification procedure, while the most active compound was isolated and characterized as tagitinin C. Tagitinin C inhibited the seedling growth of cress, *Lolium multiflorum* Lam., *Phleum pratense* L., and *Echinochloa crus-galli* (L.) P.Beauv. at concentrations greater than 0.1 mM. The concentrations required for 50% growth inhibition of tagitinin C on the roots of cress, *L. multiflorum*, *P. pratense*, and *E. crus-galli* were 0.12–0.49 mM, while those on the shoots of cress, *L. multiflorum*, *P. pratense*, and *E. crus-galli* were 0.35–0.83 mM, respectively [15]. Tagitinin C has been reported to be a major sesquiterpene lactone in *T. diversifolia* [5,14,33], and has also been found in the other species of the Asteraceae family, *Greenmaniella resinosa* (S.Watson) W.M.Sharp [34]. Sesquiterpene lactones, including tagitinin C, were reported to possess multiple biological activities, such as insecticidal, antifungal, antifeedant, antimicrobial, and cytotoxic activities [3,4,7,35]. Only a limited number of reports are available on the allelopathic substances found in *T. diversifolia* (Table 2).

A number of secondary substances in many classes have been isolated and identified from various parts of *T. diversifolia* [3,4,7]. Although these compounds have been associated with pharmacological effects, some of those compounds may possess phytotoxic activity. Phytotoxic substances in plants can be released into the soil, either by the exudates from living plant tissues or by the decomposition of plant residues, and act as allelopathic substances that inhibit germination, seedling establishment, and plant growth [36,37,38]. As described in the previous section, the root exudates and plant residues of *T. diversifolia,* and *T. diversifolia* field soils and their soil water suppressed the germination and growth of several other plant species; allelopathic substances of *T. diversifolia* may be released into the soil and the surrounding environments through the decomposition of the plant residues and the exudation of *T. diversifolia* from living plant tissues. The decomposition rate of *T. diversifolia* plant residues in soil was reported to be high. One-half of the plant residues in soils were decomposed in one week [8].

## 4. Invasive Traits of *T. diversifolia*

*T. diversifolia* is native to Mexico and Central America, but it is spreading quickly and has naturalized in more than 70 countries. The species has often been recorded as a harmful invasive plant in tropical and subtropical regions, threatening to disrupt agricultural crop production and native plant communities [2,3]. The life history characteristics, such as the high reproduction and high growth rate, as well as phenotypic plasticity of the plants, are important for the naturalization of invasive plants into non-native ranges [39,40,41]. *T. diversifolia* reproduces asexually and sexually, producing a large number (80,000–160,000 seeds/m^2^/year) of small seeds [1,12]. The phenotypic plasticity and genetic diversity of *T. diversifolia* populations were recorded to be high [42,43].

The interactions of the invasive plants with natural enemies, such as herbivores and pathogens, are also very critical for their naturalization. The high defense capacity from herbivores and pathogens contributes to the ability of invasive plants to naturalize in non-native ranges [44,45,46]. Sesquterpene lactones and flavonoids of *T. diversifolia* probably act as defensive agents against herbivores [47] and pathogens [3,4,7,35]. Insecticidal properties of the extracts and compounds of *T. diversifolia* have also been reported [10,35,48,49,50].

In addition, the interactions of the invasive plants with native plants are crucial. In fact, some invasive plants contain many phytotoxic or allelopathic substances [51,52,53]. *Centaures maculosa* Lam. is invasive and releases an allelopathic substance, catechin, which is toxic and helps its invasion into bunchgrass fields [51]. Several other observations also suggest that some invasive plant species are allelopathic, and that their allelopathic substances are more toxic against other plant species in the invasive areas than those in native areas of the invasive plants [44,53,54]. Therefore, allelopathy is probably one of the important factors for invasive plants to naturalize and establish their habitats in non-native ranges [53,54]. As describe previously, *T. diversifolia* is allelopathic, and this allelopathic property may help the invasion of this species into non-native ranges.

Many of the phytotoxic substances from the invasive plants have been reported to have multiple effects, such as antiherbivore, antifungal, antimicrobial, and allelopathic activities. The functions of these phytotoxic substances were considered to provide the plants with advantages in terms of increasing their populations in the new environments [41,53,54,55]. More than a hundred secondary metabolites have been isolated from various extracts of *T. diversifolia*, including sesquiterpenoids, diterpenoids, and flavonoids, while these compounds were also reported to possess wide ranges of biological activities [3,4,7,35]. Therefore, these compounds may enhance the competitive ability of *T. diversifolia* and make the plant invasive. The novel weapon hypothesis states that some invasive plant species gain a competitive advantage through the release of some compounds that are unique to the invading plant communities [53,54]. It is also possible that some of the compounds released from *T. diversifolia* were unique to the invaded plant communities.

## 5. Conclusions

*T. diversifolia* works as green manure, increasing crop productivity, and acts as fodder for domestic animals because of its high mineral and nutrient values [8,9,10,11]. However, the species has often been recorded as a harmful invasive plant that disturbs native plant communities [2,7,12]. The evidence summarized in this paper indicates that *T. diversifolia* is allelopathic (Table 1) and contains several phytotoxic substances, such as sesquiterpene lactones (Table 2). The evidence also suggests that some of the phytotoxic substances in *T. diversifolia* are probably released into the soil through the decomposition of the plant residues and the exudation from living plant tissues of *T. diversifolia*, which act as allelopathic substances. The allelopathic substances can inhibit the germination and growth of neighboring plants [38,52,53,54]. Therefore, the allelopathic substances released from *T. diversifolia* may provide the plants with a competitive advantage against native plants, and may contribute to the plants establishing their habitats as invasive plant species. Allelopathy of *T. diversifolia* may be involved in the invasive potential of *T. diversifolia.*

## Figures and Tables

**Table 2:** Allelopatic substances found in *T. diversifolia* and target plant species for their activity.

Allelopathic Substance	Phytochemical Class	Inhibitory Activity	Target Plant Species and Reference
Tagitinin A	Sesquiterpene lactone	Germination	Radish, cucumber, onion [32]
		Growth	Wheat, tomato, onion, lettuce, cress [14]
Tagitinin C	Sesquiterpene lactone	Germination	Radish, cucumber, onion [32]
		Growth	Wheat, tomato, onion, lettuce, cress [14]
		Growth	Cress, *Lolium multiflorum*, *Phleum pratense, Echinochloa crus-galli* [15]
Tagitinin F-3-*O*-methyl ester	Sesquiterpene lactone	Growth	Wheat [14]
3-Methoxytirotundin	Sesquiterpene lactone	Growth	Wheat [14]
Tirotundin	Sesquiterpene lactone	Growth	Wheat [14]
1β-Methoxydiversifolin	Sesquiterpene lactone	Growth	Wheat, tomato, onion, lettuce, cress [14]
3β-Acetoxy-8β-isobutyloxyreynosin	Sesquiterpene lactone	Growth	Wheat [14]
3β-Acetoxytithifolin	Sesquiterpene lactone	Growth	Wheat [14]
3α-Acetoxycostunolide	Sesquiterpene lactone	Growth	Wheat [14]
8β-Isobutyryloxycumambranoide	Sesquiterpene lactone	Growth	Wheat [14]
2-Formyl-4-hydroxy-4α-methyl-3-(3-oxobutyl)cyclohexaneacetic acid	Sesquiterpene lactone	Growth	Wheat [14]
(2*E*,6*E*10*E*)-3-(hydoxymethyl)-7,11,15-trimethylhexadeca-2,6,10,15-tetraene-1,14-diol	Diterpene	Growth	Wheat [14]
Hispidulin	Flavone	Germination	Radish, cucumber, onion [32]
